# Salt tolerance involved candidate genes in rice: an integrative meta-analysis approach

**DOI:** 10.1186/s12870-020-02679-8

**Published:** 2020-10-01

**Authors:** Raheleh Mirdar Mansuri, Zahra-Sadat Shobbar, Nadali Babaeian Jelodar, Mohammadreza Ghaffari, Seyed Mahdi Mohammadi, Parisa Daryani

**Affiliations:** 1 Department of Systems Biology, Agricultural Biotechnology Research Institute of Iran (ABRII), Agricultural Research, Education and Extension Organization (AREEO), PO Box 31535-1897, Karaj, Iran; 2 Faculty of Crop Science, Department of Plant breeding and Biotechnology, Sari Agricultural Science and Natural Resources University, Sari, Iran

**Keywords:** Meta- analysis, RNA-seq, Microarray, QTLs, Salinity stress, *Oryza sativa*

## Abstract

**Background:**

Salinity, as one of the main abiotic stresses, critically threatens growth and fertility of main food crops including rice in the world. To get insight into the molecular mechanisms by which tolerant genotypes responds to the salinity stress, we propose an integrative meta-analysis approach to find the key genes involved in salinity tolerance. Herein, a genome-wide meta-analysis, using microarray and RNA-seq data was conducted which resulted in the identification of differentially expressed genes (DEGs) under salinity stress at tolerant rice genotypes. DEGs were then confirmed by meta-QTL analysis and literature review.

**Results:**

A total of 3449 DEGs were detected in 46 meta-QTL positions, among which 1286, 86, 1729 and 348 DEGs were observed in root, shoot, seedling, and leaves tissues, respectively. Moreover, functional annotation of DEGs located in the meta-QTLs suggested some involved biological processes (e.g., ion transport, regulation of transcription, cell wall organization and modification as well as response to stress) and molecular function terms (e.g., transporter activity, transcription factor activity and oxidoreductase activity). Remarkably, 23 potential candidate genes were detected in *Saltol* and hotspot-regions overlying original QTLs for both yield components and ion homeostasis traits; among which, there were many unreported salinity-responsive genes. Some promising candidate genes were detected such as pectinesterase, peroxidase, transcription regulator, high-affinity potassium transporter, cell wall organization, protein serine/threonine phosphatase, and CBS domain cotaining protein.

**Conclusions:**

The obtained results indicated that, the salt tolerant genotypes use qualified mechanisms particularly in sensing and signalling of the salt stress, regulation of transcription, ionic homeostasis, and Reactive Oxygen Species (ROS) scavenging in response to the salt stress.

## Background

Currently, rice ranks as the most important food crop in the world before wheat and maize supplying a major source of calorie for more than 3.5 billion people all over the world [1, 2]. However, rice is classified as a very sensitive crop to salinity in both seedling and reproductive stages, while excess salt in soil is one of the most widespread abiotic stresses in Asia and some river deltas in Europe [3, 4]. Salinity challenge at the seedling stage causes the growth arrest or death of rice plant, that reduces significantly the yield [5, 6]; therefore, increasing the salinity tolerance at the seedling stage would be effective to improve the environmental adaptation and yield maintenance in rice. It is necessary to understand the mechanisms underlying the salinity stress tolerance because of increasing the population, limited arable land, and climate changes that can provide us a better perspective regarding how to manage the increasing demand for high-yielding rice [2, 7]. Salinity tolerance is a complicated trait both genetically and physiologically [8]. Rice, as a well-studied model organism, is particularly rewarding to investigate the salinity stress responses [7]. Many QTLs have been eventually identified in the rice breeding programs [9–16], including a major locus on chromosome 1, namely *Saltol*, involved in Na/K homeostasis derived from Pokkali and SKC1 (OsHKT1; 5) from Nona Bokra [17]. Isolation of the identified QTLs related to salt tolerance can be highly beneficial to improve the global agriculture and food security but it is also a challenging task [18]. Although, many QTLs have been found but there is still limited knowledge regarding the salinity tolerance-related gene networks in rice. Technologies such as microarray and gene expression profiling based on sequencing approaches accelerate the progress toward a comprehensive understanding of the genetic mechanisms related to responses to environmental stresses [19, 20]. Fast advances and decreased price of high-throughput sequencing technology have led to extensive application of RNA sequencing in various species in the recent years [21]. Therefore, many differentially expressed genes (DEGs) have been identified among the contrasting samples through mentioned technologies. Researchers have recently used an integration of DEGs and QTLs as a confident method to identify the potential candidate genes [22]. Currently, a great and varied set of genomic data has become publicly available; subsequently, a combination of numerous accessible data can rise the consistency and generalizability of the results. Combining the results obtained from the independent but associated studies is called “meta-analysis (MA)”; thus researchers can obtain more exact estimation regarding the differential gene expression by increasing the statistical power in MA [23, 24]. Breeding by introgression of the identified QTLs is restricted owing to the conflict of QTLs in different genetic backgrounds and environments [25]; while meta- QTL analysis suggests a chance to use QTL data from various mapping populations with diverse genetic backgrounds to detect the accurate position of the QTLs [26]. Several studies have identified the accurate meta-QTLs of with various traits for mining the candidate genes in rice and other crop plants [26–30]. However, an integrative meta-analysis approach was employed in this study that resulted in finding several promising genes involved in salinity tolerance, among which, some of the important genes/gene families with sufficient evidence are listed and discussed later to support their candidacy in the rice. All data produced in the previous studies were used to identify the rice candidate genes related to salt tolerance and then, the candidate genes were confirmed using the meta-analysis. Findings of this study provide valuable information on the genes and pathways involved in salinity tolerance in rice.

## Results

### Salinity tolerance associated Meta-QTLs in rice

**Fig. 1:**
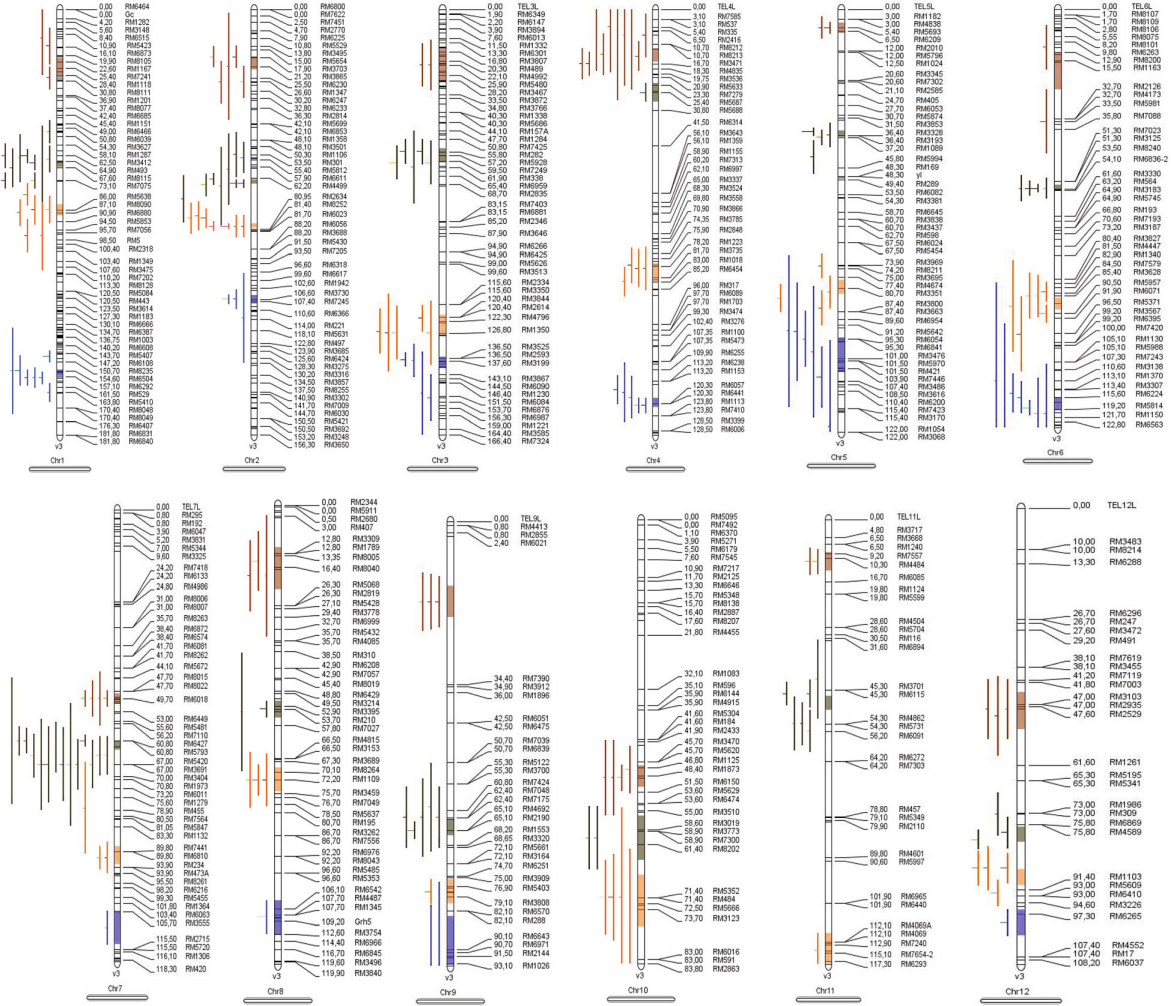
Meta-QTL positions for traits associated with the salt tolerance (Table S1) on 12 chromosomes of rice. Vertical lines on the left of the chromosomes show the confidence interval of each QTL. Marker names and positions (in cM on the consensus map) are indicated on the left. The colors indicate Meta-QTL positions for traits associated with the salt tolerance

A total of 265 QTLs related to 32 traits were collected in this study using the Simple Sequence Repeats (SSR) markers (Table S1, S2) among which, 126 and 139 QTLs were selected for further analysis in normal and salinity conditions (Table S3). Most of the QTLs belonged to the salinity tolerance score (STS) (27 QTLs), shoot potassium concentration (KS) (26 QTLs), shoot sodium concentration (NS) (21 QTLs), chlorophyll content (CHL)(19 QTLs) and shoot dry weight (DSW) traits (19 QTLs) (Fig.S1). In contrast, the rare QTLs belonged to the number of sterile spikelets (NSS) [20], dead seedling rate (DSR), leaf potassium concentration (KLV), reduction of seedling height (RSH) and reduction of leaf area (RLA) traits (Fig.S1). The highest number of QTLs were observed on chromosome 1 (37 QTLs) and 2 (36 QTLs) followed by chromosome 7 (29 QTLs), while chromosome 8 (12 QTLs) and 11 (12 QTLs) had the lowest number of QTLs (Fig.S2). The phenotypic variance described by the original QTLs was different from 0.7 to 33.25% and the confidence interval (CI) of markers was different from 0.99 to 84.36 cM (Table S3). After the integration of all the collected QTLs on the consensus map, 46 meta-QTLs were identified in 12 chromosome of rice (Fig. 1). There were meta-QTLs with a CI of 95% based on the lowest Akaike information criterion (AIC) values. Remarkably, second meta-QTLs on Chr7: M-QTL2, Chr2: M-QTL2, and Chr1: M-QTL2 included the highest number of initial QTLs (17,16 and 12, respectively), which covered a relatively narrow CI (4.78, 1.82 and 2.84 cM, respectively) (Table S4). These meta-QTLs support the important traits; for example, ratio of the shoot sodium and potassium concentration (NKS), number of fertile spikelets (NFS), root length (RTL), and chlorophyll content (Table S4). Chr12: M-QTL4, Chr 9: M-QTL3 and Chr3: M-QTL2 had the highest mean percentage of phenotypic variation (*R*^*2*^), which can be considered as the main effective QTLs for the involved traits (Table S4). A total of 9366 genes were detected in 46 meta-QTL positions, among which, Chr8: M-QTL2 contained the highest number of genes (868 genes); while, Chr12: M-QTL2 contained the lowest number of genes (14 genes) (Table S4). Moreover, the proportion of functionally characterized annotated genes (27%) is actually limited compared to the about 73% of unannotated genes with allocated putative functions. It is intersting to note that, 81 genes were identified on Chr1: M-QTL2 which were located in *Saltol* region.

### Expression profiling analyses in the salinity tolerant genotypes of rice

The DEGs were identified under salinity stress compared to control conditions in the salinity tolerant genotypes. A total of 1714 DEGs were observed in the roots of FL478 as a salinity tolerant genotype, among which, 927 and 787 were up- and down-regulated in the salinity conditions [31]. DEGs from multiple RNA-seq datasets were combined and the DEGs were classified into root, shoot, seedling, and leaves to have a deeper understanding about the salt responsive genes in the salinity tolerant rice genotypes. A total of 3030, 396, 703 and 723 DEGs were merely identified in root, shoot, seedling and leaves, respectively (Fig.S3). Also, raw microarray data from nine independent experiments were downloaded (Table S5) and analyzed uniformly. Microarray meta-analysis suggested 11,694 DEGs, among which, 4121, 13, 6247 and 1199 DEGs were exclusively expressed in root, shoot, seedling and leaves, respectively (Fig.S4). In addition, a total of 4763 and 5862 DEGs were merely up- and down-regulated, respectively, in the salinity tolerant genotypes.

### Integration of DEGs from two Meta-analysis approaches

Identified DEGs in both RNA-Seq and microarray meta-analysis were combined to confirm the consistency of the obtained results. A list of overlapping DEGs were detected in four tissues, separately after removing all the duplicate genes.

**Fig. 2:**
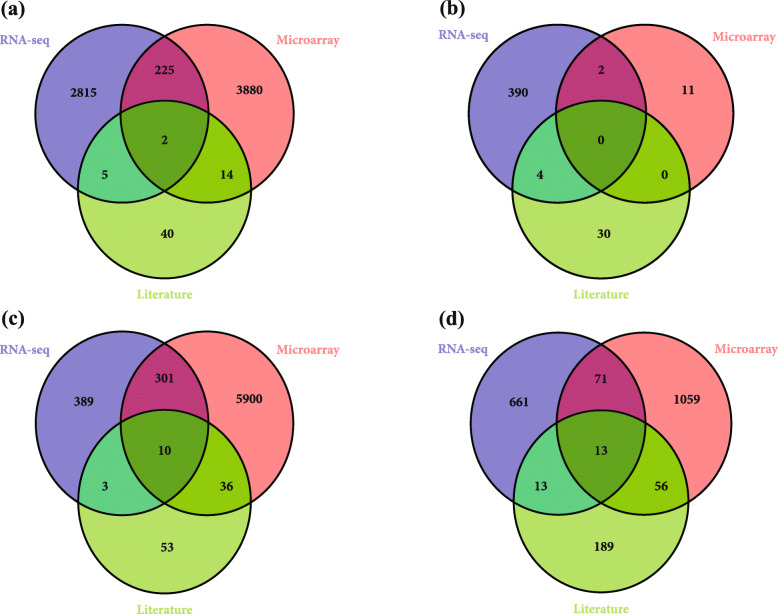
The results of comparison between differentially expressed genes under salt stress conditions in the tolerant genotypes revealed by RNA-Seq and microarray data analysis, or through literature review in (**a**) root, (**b**) shoot, (**c**) seedling and (**d**) leaves

Comparative transcriptome analysis indicated that 227, 2, 311, and 84 DEGs were commonly detected by the RNA-Seq and microarray respectively in root, shoot, seedling, and leaves tissues (Fig. 2). A total of 4255 and 10,980 DEGs were merley identified by the RNA-Seq and microarray meta-analysis, while only 156 DEGs were previously reported in the literature (Fig. 2).

### Detection of the DEGs in the meta-QTL positions

**Fig. 3:**
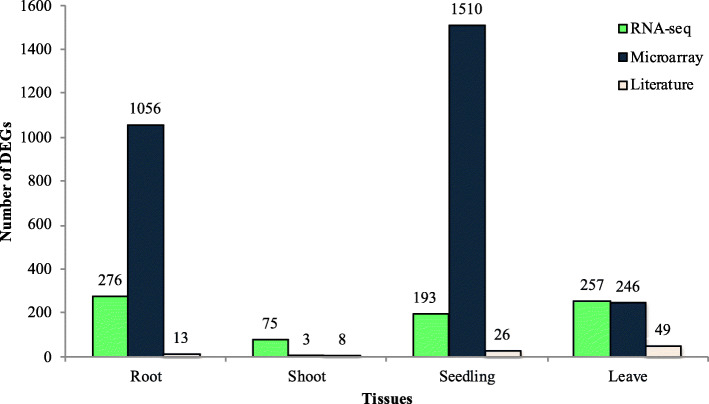
The number of differentially expressed genes identified by RNA-Seq and microarray data analysis, or through literature review, which are located on the meta-QTL positions in each tissue (roots, shoots, seedlings, and leaves)

There were a total of 1345, 86, 1729, and 552 DEGs in the meta-QTL positions in root, shoot, seedling and leaves, respectively (Fig. 3). Among the identified DEGs in the meta-QTL positions, 664 and 2359 DEGs were identified by the RNA-Seq and microarray meta-analysis, respectively while, only 82 DEGs located in the meta-QTL positions were previously reported in the literature (Fig. 3).

### Functional annotation of DEGs located in the meta-QTL positions

Gene ontology enrichment analysis was performed to determine the biological roles of the DEGs located in the meta-QTL positions. Carbohydrate metabolic process, regulation of cellular process, regulation of transcription, response to stress and regulation of nitrogen compound metabolic process were indicated as dominant terms in the biological processes (BP) (Fig.S5). Moreover, some BP terms including regulation of transcription, inorganic anion transport, anion transport, ion transport as well as regulation of gene expression, cell wall organization and modification were significantly enriched (Fig.S5). The most significant over-represented molecular function (MF) terms were nucleotide binding, ATP binding, anion transmembrane transporter activity, inorganic anion transmembrane transporter activity, transcription factor activity and oxidoreductase activity (Fig.S5). In terms of cellular component (CC) ontology, the most significant enriched terms were intrinsic to membrane and integral to membrane (Fig.S5).

### Mining the potential candidate genes in the meta-QTL positions

Exploring the meta-QTL regions for the common genes were resulted in finding 60 potential candidate genes in the root (Table S6), among which, only four genes were previously reported associated to the salinity response. Remarkably, LOC_Os01g20980.1 (coding Pectinesterase) was found in Chr1: M-QTL2 located in *Saltol* region (Table S6). Ion homeostasis related QTLs were also found in Chr1: M-QTL2 which controling the KLV, NS, NKS, KS and RN traits (Table S4). Overall, identified potential candidate genes were classified into several terms in the root tissue, for example, transcription factor (e.g., *TIFY*, *GRAS*, *HOX*, *WRKY* and *MYB* family), signaling (e.g., *OsWAK125*, *pectinesterase*,*OsMKK1*, and *CHIT15*), transporter (e.g., *OsHKT1* and some genes coding transmembrane transport and anion transporter) and some other functions (e.g., *NUDIX* family, genes coding the aspartic protease) (Table S6).

Four genes in meta-regions on Chr2, 3, and 8 were identified as potential candidate genes in the shoot, as discussed in the literature; for instance, *TIP2–1* (LOC_Os02g44080.1) in Chr2: M-QTL4 (Table S6). Chr2: M-QTL4 was integrated with seven initial QTLs controlling RTL and some other related traits (e.g. S, KS, NKS, SIS, and NS) (Table S4). Moreover, two transcription factors (LOC_Os03g08310.1 and LOC_Os08g15050.1) were identified respectively as possible candidate genes in Chr3: M-QTL1 and Chr8: M-QTL2 (Table S6) supporting the root length and photosynthesis related traits, respectively (Table S4). It is interesting to note that, LOC_Os03g08310.1 (coding *TIFY11A*) was identified as common candidate gene in the root and shoot (Table S6).

Our results indicated 98 potential candidate genes in the seedling including 84 DEGs located in the M-QTLs that were not reported yet. However, 14 genes have been already considered in the literature (Table S6). Functional classification of these potential candidate genes further suggested that they were related to the transcription regulation (e.g., *AP2*, *WRKY*, *HOX*, and *GRAM* family), signal transduction (e.g., *CIPK24*, *GDSL*) and there were some genes with another functions including kinase, phosphatase, and transporter terms under salinity stress in seedling tissue (Table S6). Remarkably, LOC_Os01g20830.1 (coding a transporter protein) and LOC_Os01g21144.1 (with unknown function) were found in *Saltol* region on Chr1: M-QTL2 (Table S6). As well, there were some potential candidate genes in hotspot-regions; for example, *WRKY70* (LOC_Os05g39720.1) in Chr5: M-QTL4 and *PP2C* (LOC_Os06g48300.1) in Chr6: M-QTL4 (*R*^*2*^ = 10.31%) (Table S4, S6). Moreover, some genes were identified as potential candidate genes in Chr2: M-QTL1, Chr8: M-QTL1, Chr10: M-QTL3, and Chr11: M-QTL1; these meta-regions were integrated the importance of the initial QTLs for photosynthesis, straw dry weight, yield components (e.g. QGW, DF and NFS) and RTL traits (Table S4, S6).

Totally, 28 potential candidate genes were identified in the leaves among which, 14 genes were found in the literature. The LOC_Os01g22249.1 (coding the peroxidase) located in *Saltol* region in Chr1: M-QTL2 was identified as another leading candidate gene. Notably, *OsHKT1* (LOC_Os06g48810.1) and *PP2C* (LOC_Os06g48300.1) were found in the hotspot-regions in Chr6: M-QTL4 (Table S4, S6).

**Fig. 4:**
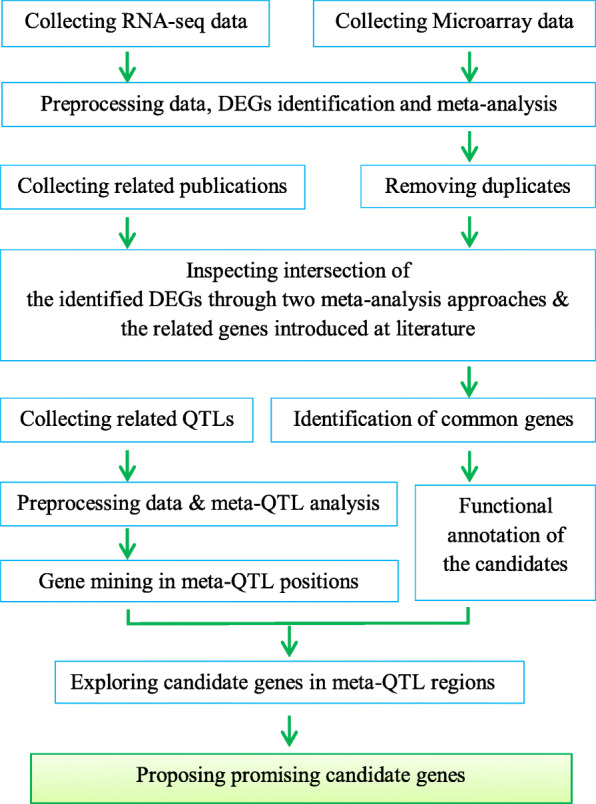
Flowchart showing different steps of meta-analysis pipeline used to identify the promising candidate genes involved in the salinity tolerance. The differentially expressed genes detected by more than one approach called common genes in this manuscript. To find the potential candidate genes, the common genes were sought in the salinity tolerance associated meta-QTLs regions. The potential candidate genes that were located on hotspot-regions overlying original QTLs for both yield components and ion homeostasis traits were assumed as promising candidate genes

The obtained results indicated that, 20 genes were located on the hotspot-regions containing original QTLs for both yield components and ion homeostasis traits which could be suggested as promising candidate genes (Fig. 4, Table 1). The promising genes were related to the following functions: pectinesterase, peroxidase, transcription regulation, high-affinity potassium transporter, protein serine/threonine phosphatase, cell wall organization and a CBS domain containing gene, among which, there were 2 genes in *Saltol* region (Table 1).

**Table 1:** The promising genes associated with salinity tolerance. The differentially expressed genes detected by more than one approach (common genes) and located on meta-QTLs regions overlying original QTLs for both yield components and ion homeostasis traits were assumed as promising candidate genes in this study (the pipeline is presented in Fig. 4)

Gene ID	Gene name/ function	Meta position	Tissue (Expressed in)
LOC_Os01g20980.1	Pectinesterase	Chr1: M-QTL2	Root
LOC_Os01g22249.1	Peroxidase	Chr1: M-QTL2	Leaves
LOC_Os02g06410.1	CBS domain containing membrane protein	Chr2: M-QTL1	Root
LOC_Os02g06640.1	Ubiquitin family protein, putative, expressed	Chr2:M-QTL1	Leaves
LOC_Os04g03810.1	*OsSub38*, Putative Subtilisin homologue, expressed	Chr4:M-QTL1	Root
LOC_Os04g26870.1	Oxidoreductase, aldo/keto reductase family	Chr4: M-QTL2	Seedling
LOC_Os04g06910.1	Expressed protein	Chr4:M-QTL1	Seedling
LOC_Os04g10750.1	Inorganic phosphate transporter	Chr4:M-QTL1	Seedling
LOC_Os05g42130.1	*MOC1*,Transcription regulation, GRAS family	Chr5: M-QTL4	Root
LOC_Os05g39720.1	*WRKY70,* Transcription regulation, Negative regulator of stomatal closure through SA- and ABA-mediated signaling	Chr5: M-QTL4	Seedling
LOC_Os05g39770.1	Aminotransferase, putative, expressed	Chr5:M-QTL4	Leaves
LOC_Os05g38660.1	Expressed protein	Chr5:M-QTL4	Seedling
LOC_Os05g40010.1	*LTPL17*, Protease inhibitor/seed storage/LTP family protein precursor, Signal domain	Chr5:M-QTL4	Seedling
LOC_Os05g41670.1	Expressed protein	Chr5:M-QTL4	Seedling
LOC_Os05g39990.1	Plant-type cell wall organization	Chr5:M-QTL4	Root
LOC_Os05g39250.1	Phosphatidylethanolamine	Chr5:M-QTL4	Root
LOC_Os06g48860.1	*OsSAUR28*, Auxin-responsive SAUR gene family member, expressed	Chr6:M-QTL4	Root
LOC_Os06g48810.1	*OsHKT1*, Na^+^ transporter, k^+^ transporter,cation transmembrane transporter activity	Chr6: M-QTL4	Root and Leaves
LOC_Os06g48300.1	*PP2C*, protein serine/threonine phosphatase activity	Chr6: M-QTL4	Root, Seedling & leaves
LOC_Os06g49190.1	*LTPL154*, Protease inhibitor/seed storage/LTP family protein precursor, Signal domain	Chr6:M-QTL4	Seedling

### Validation of differential gene expression using qRT-PCR

**Fig. 5:**
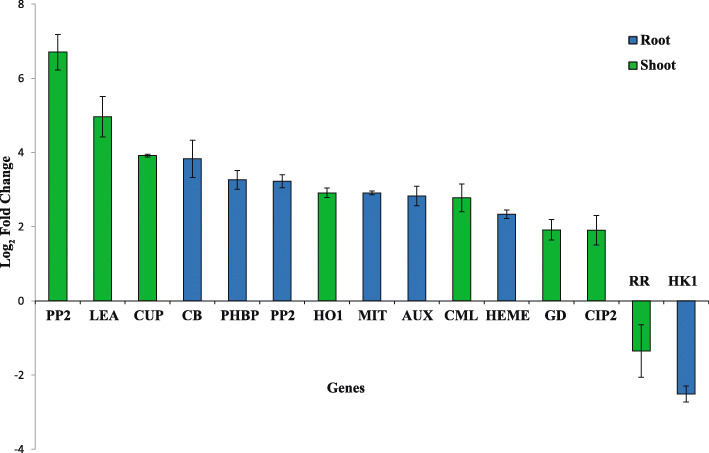
Validation of selected genes using qRT-PCR in root and shoot tissues of FL478 (tolerant genotype). Bar graphs depict the relative transcript abundance of the selected potential candidate genes in FL478 under different conditions. Data points are represented as log2 fold change values

To further validate the potential candidate genes, 15 genes were selected for qRT-PCR in FL478 as a salt tolerant genotype (Fig. 5). The qRT-PCR results were confirmed the outcome of the meta-analysis (Fig.S6).

## Discussion

**Fig. 6:**
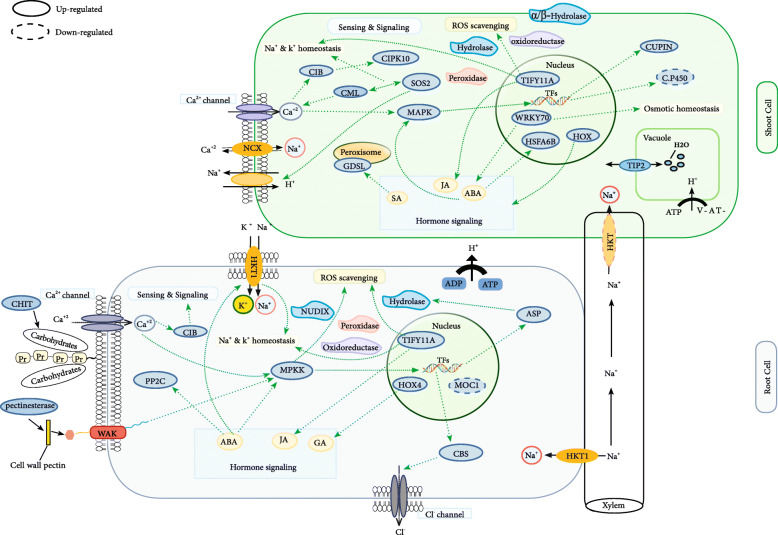
The schematic representation of the molecular response to salt stress in the tolerant genotypes. Some candidates are depicted, whose coding gene was differentially expressed under the salt stress conditions located on the meta-QTLs

Rice is highly influenced by the salinity stress at seedling and reproductive stages. High salinity concentrations lead to the ionic imbalances, dehydration, osmotic stress, and oxidative damage. Therefore, it is important to identify the most accurate QTLs and the involved candidate genes. Herein, a panel of potential candidate genes both located on the meta-QTL regions and differentially expressed ones in the salinity stress conditions was provided in the tolerant genotypes (Fig. 6).

### Sensing and signaling

Tolerance of the plants against the abiotic stresses including salinity is activated by the complex multicomponent signaling pathways to return the cellular homeostasis and promote the survival [32]. The plant cell wall is one of the first layers for biotic and abiotic stimuli perception, and cell wall remodeling provides a general response mechanism to stresses [33]. Here were several genes coding integral components of membrane and cell wall organization in the hotspot-regions. *OsWAK125* was found in Chr12: M-QTL1 and up-regulated in the roots (Table S6, Fig. 6), belonging to the wall-associated kinase family and has been mainly investigated as a potential candidate for the cell wall “sensor” [34, 35]. The Wall Associated Kinases (WAKs) firmly bind to the pectic network of the cell wall, protrude the membrane, and link it to the cytoplasm where a Serine/ Threonine (Ser/Thr) kinase domain is responsible for further signaling [34, 35]. A drought and salinity responsive class of cell wall-related genes (represented by the pectinesterase) was also found in *Saltol* region up-regulated in the roots (Table S6, Fig. 6). Various crops such as soybean, wheat, and tomato have been shown to have higher levels of pectin remodeling enzymes in tolerant cultivars than susceptible genotypes under salinity and drought stress [33]. Several Ser/Thr phosphatase genes were differentially expressed in the leaves at seedling stage in the hotspot-regions (Table S6). Ser/Thr phosphatases play significant roles in the regulation of the adaptive stress responses and signaling pathways in various crops such as potato, wheat, and rice [36–40].

*OsMKK1* in Ch6: M-QTL12 and *OsCHIT15* in Chr10:M-QTL3 were also detected, which up-regulated in the roots, and mediating the salinity signaling in rice (Table S6, Fig. 6) [41]. Plant chitinases play an important role in the response to abiotic stress; it has also been reported that hydrolysis of the carbohydrate chains by the chitinases indicates its possible role in signaling or osmotic adjustment functions [34]. Moreover, seven hydrolase coding genes involved in the signaling pathways were among the DEGs located on the meta-QTL regions (Table S6), among which two GDSL-like lipase/acylhydrolase enzymes in Chr5:M-QTL2 and Chr6:M-QTL1 were up-regulated in the seedlings under salinity stress (Table S6, Fig. 6). Furthermore, *OsCIPK24* (SOS2) in Chr6:M-QTL3 and *OsCIPK10* in Chr3:M-QTL2 were up-regulated in the seedlings (Table S6, Fig. 4). CIPK (CBL- Interacting Protein Kinases) pathway has emerged as a main signaling pathway and adjusts the salt tolerance in rice [42, 43]. A generic signal transduction pathway starts with signal perception, followed by the generation of the second messengers)e.g., inositol phosphates and Reactive Oxygen Species (ROS)) and the transcription factors controlling the specific sets of stress-regulated genes [44].

### Transcription regulation

Transcription factors are important for emergence of any phenotype, as they are able to regulate the expression of all the related genes [32]. *HSFA6B* (located in Chr1:M-QTL3, up-regulated in the seedlings) acts as a positive regulator downstream of Abscisic Acid (ABA) signaling directly bound to the promoter of Dehydration-Responsive Element-Binding (DREB) and increasing its expression (Table S6, Fig. 6). Upregulation of the Dehydration-Responsive Element -Binding protein 2A (DREB2A) can activate the various genes related to stress tolerance in different plant species [45]. It has also been reported that over-expression of *OsTIFY11* (located in Chr3:M-QTL1, up-regulated in the shoot and root) increased the tolerance to salinity stress through the Jasmonic Acid (JA) signaling and through modulating the potassium homeostasis (Table S6, Fig. 6) [46]. There were *OsHOX22* and *OsHOX24* from homeobox familyin Chr3:M-QTL1 and Chr4:M-QTL3, respectively, which were both up-regulated in the seedlings (Table S6, Fig. 6). *OsHOX24* was the most up-regulated gene under 150 mM NaCl in the salt tolerant genotype (FL478); while it was highly down-regulated in the salt sensitive genotype (IR29) [31]. Also, the role of *OsHOX24* has been already found to regulate the abiotic stress responses through fine tuning the expression of stress-responsive genes in rice [47]. Moreover, there was *OsWRKY70* in Chr5:M-QTL4 and up-regulated in the seedlings (Table S6, Fig. 6). It has been stated that *OsWRKY70* as a negative regulator of stomatal closure through SA- and ABA-mediated signaling, play important role in the plant tolerance to osmotic stress [48]. Moreover, GRAS (located in Chr5:M-QTL4 and down-regulated in the roots) proteins belong to a plant-specific transcription factor family involved in many plant processes including plant growth and development as well as abiotic stress responses (Table S6, Fig. 6) [49, 50]. It has also been reported that *MOC1* encodes a nuclear transcription factor from GRAS family. *MOC1* acts as a positive regulator of lateral branching or increased tiller number [51].

### ROS inhibition

One of the key mechanisms to increase the plants adaptation to detrimental environmental conditions including high salt concentrations is regulation of the toxic ROS levels [33, 52]. Nudix hydrolase was found in Chr4: M-QTL3 and was up-regulated in the roots (Table S6, Fig. 6), generally removing the excess toxic metabolites or controlling the accessibility of intermediates in the metabolic pathways [53]. Also, there was a peroxidase coding gene belongs to the antioxidant system in Chr1:M-QTL2 that was up-regulated in the leaves (Table S6, Fig. 6). Transgenic Arabidopsis plants expressing the cytosolic peroxidase genes have been reported to show higher salt tolerance [20]. In addition, there was a hydrolase coding gene belonging to the alpha/beta fold family domain containing protein in Chr3:M-QTL3that was up-regulated in the seedling (Table S6, Fig. 6). It has been reported that overexpression of a gene coding α/β-hydrolase fold enzyme led to significantly higher salinity tolerance compared to the wild-type because of protecting the membrane integrity and increasing the ROS scavenging capacity in the sweetpotato [54].

### Ionic homeostasis

Regulation of the ion flux under salinity stress is necessary for the cells to keep the concentrations of toxic ions at low levels and to collect the essential ions. Salinity stress up-regulates the trasporter encoding genes such as Na^+^ and K^+^ transporters and vacuolar Na^+^/H^+^ exchangers [55]. Several transporters were observed in the meta-QTL positions among which, *HKT1* was found in Chr6: M-QTL4; down-regulated in the leaves and up-regulated in the roots (hotspot-region, Table S6, Fig. 6). High affinity K^+^ transporter known as Na^+^/K^+^ co-transporters reduces the transport of Na^+^ to the shoots and positively regulate the salinity tolerance in rice and Arabidopsis [56]. Two genes encoding the vacuolar protein with signal peptide domain were identified in Chr1: M-QTL3 and Chr3:M-QTL2 (Table S6); up-regulated in the seedling. The genes coding the sodium/calcium exchanger (NCX) in Chr12:M-QTL4; up-regulated in seedling (Table S6, Fig. 6), which play significant roles in Ca^2+^ signaling and ion homeostasis. Sodium/calcium exchangers use the Na^+^ electrochemical gradient through the plasma membrane to extrude the intracellular Ca^2+^ [57, 58].

### Other salt tolerance related potential candidate genes

Twenty three unknown potential candidate genes were found among which, five genes possess the CBS or cupin domain(s) in their sequence. For instance, a gene containing CBS domain was located in Chr2:M-QTL1 that up-regulated in the roots. Previous reports have indicated that, it plays a role in the salinity and oxidative stress tolerance through influencing the chloride channels (Kushwaha et al. 2009). It has also been reported that overexpression of *OsCBSX4* improved the tolerance against salinity and oxidative stress in tobacco transgenic lines [59].

Furthermore,four genes possessing the cupin domain(s) in their sequence were found in various M-QTL positions (Table S6) while there were up-regulated in the seedlings. According to the previous reports, cupin domain might play a role in improving the seed germination in rice under salinity stress because the proteins having the cupin domain(s) were observed near the position of QTLs related to the seed dormancy, seed reserve utilization, and seed germination [60].

## Conclusions

To inspect the molecular mechanisms by which tolerant genotypes respond to the salinity stress, we employed an integrative approach to identify candidate genes related to salt tolerance in rice. The obtained results indicated that, the salt tolerant genotypes utilize more effective mechanisms in response to the salt stress (Fig. 6) particularly in terms of 1) Sensing and signalling of the salt stress; several genes coding the cell wall organization*,* pectinesterase, Ser/Thr phosphatase, chitinase, CIPKwere observed in the hotspot-regions that were differentially expressed in the tolerant genotypes. 2) Regulation of transcription; several salinity responsive transcription factors (TFs) belonging to different families including TIFY, MYB, HSF, HOX, WRKY, AP2, and GRAS families were found both in the meta-QTL regions and among the DEGs, which have been shown to play essential roles in the salinity tolerance in rice. 3) Ionic and osmotic homeostasis; some transporters were also among the promising candidate genes such as *HKT1* (Na/ K transporter)*,* NCX (sodium/calcium exchanger), and TIP2–1 (aquaporin). 4) ROS scavenging; there were many important genes involved in detoxification such as hydrolase, oxidoreductase, and peroxidase among the DEGs that were located in the meta-QTL positions. Further research on these promising candidate genes can bring about beneficial information which would be used to improve salt tolerance in the given genotypes through genetic engineering or molecular breeding.

## Methods

### Meta-analysis of QTLs

#### Preparing the QTL data

All the reported QTLs related to the salinity tolerance in rice (from 2009 to 2018) were collected including those identified in 15 previously published studies [9–14, 16, 61–68]. The QTLs data including the parental lines, the type and size of QTL mapping population, and the number of QTLs per trait were provided. Moreover, the flanking molecular markers, Confidence Interval (CI), QTL position, Logarithm of the Odds (LOD) score, and Proportion of Phenotypic Pariance Explained (PVE or R2) were evaluated with respect to each QTL. The QTLs used in this study were derived from various population types (including: F2, backcrossed lines (BC3F4), Recombinant Inbred Lines (RILs)), and sizes (from 87 to 285 plants) from different tissues at seedling and reproductive developmental stages (Table S1).

#### Consensus map and QTL projection

The consensus QTL regions were identified using the BioMercator software [69]. The map of the International Rice Microsatellite Initiative (IRMI) available at https://archive.gramene.org (IRMI_2003) was used as the reference map for Meta-QTL analysis. The 95% CI of the initial QTL was computed using the following formulas before projecting the QTLs on the consensus map:

(i) For F2 lines: $$ CI=\frac{530}{N\times {R}^2} $$
(ii) For Double Haploid (DH) lines: $CI=\frac{287}{N\times {R}^2}$
(iii) For RILs: $CI=\frac{163}{N\times {R}^2}$

Where, N is the population size and *R*^*2*^ is the percentage of phenotypic variation explained by the related QTL. The scaling rule between the marker intervals of the initial QTLs was used for the QTL positions on the consensus chromosome map.

#### Meta-analysis of the QTLs

Meta analysis was performed by the default parameter sets in the BioMercator V4.2 tool. The consensus QTL was calculated as 1, 2, 3, and n models by the software. The Akaike Information Criterion (AIC) was used to select the QTL models on each chromosome [70]. According to the AIC value, the QTL model with the lowest AIC value was considered as a significant model.

### RNA –sequencing

RNA-Seq data was obtained from our previous study on two contrasting genotypes of *Oryza sativa* under salinity stress [31]. Briefly, the young seedlings of FL478 (Salt tolerant) and IR29 (Salt sensitive) were treated with 150 mM NaCl and the root samples were collected 24 h after inception of the salt stress. Along with, normal samples (at the same conditions but without salinity treatment) were also collected as control samples [31]. The purified RNA was used to construct the cDNA library; the qualified libraries were subsequently sequenced using IlluminaHiSeq™ 2500 sequencer. The transcriptome raw data including control (SRR7944745 and SRR7944784) and salt treated samples (SRR7944792 and SRR7944793) of FL478, and control samples (SRR7945188 and SRR7945229) and salt treated samples (SRR7945230 and SRR7945234) of IR29 are available at SRA (Sequence Read Achieve) of NCBI database. The quality of datasets was conducted using the FastQC tool [71]. TopHat was used to map eight paired-end sequencing libraries of two rice genotypes against the rice reference genome sequences IRGSP 1.0 (ftp://ftp.ensemblgenomes.org/pub /plants) [71]. Raw sequencing reads were then assembled through Cufflinks and Cuffmerge meta assembler utilities [71]. Finally, DEGs were identified by Cuffdiff utility, with log2 fold change ≥ 1 (up-regulated genes) and ≤ (− 1) (down-regulated genes) and *Q-*value cut-off of ≤0.05.

### Meta-analysis of the gene expression data according to tissues

#### RNA-seq Meta-dataset

The available (by the time of this analysis) transcriptome datasets of rice plants exposed to salinity stress were collected from the National Center for Biotechnology (NCBI) database (Table S7). The genes with − 1 ≥ log2 fold change ≥ 1 and significant Q-value (FDR ≤ 5%) were considered as DEGs from these RNA-seq datasets and were classified into four tissues (i.e. shoot, root, seedling, and leaves).

#### Microarray Meta-analysis

Rice expression data subjected to salt stress were obtained from the NCBI’s Gene Expression Omnibus repository (GEO) [71, 72]. Totally, nine GEO datasets were downloaded from the affymetrix platform Rice Genome Array (Affymetrix or Agilent microarray platforms) (Table S5). Each set of the expression data was preprocessed separately. The LIMMA package in the R program was used to analyze Agilent microarray data [73], while affymetrix platforms were handled in the R program by the Affy package. The raw data of each source was preprocessed by the quantile normalization and Robust Multi-Array Average background correction. Then, the probes with low-intensity and non-informative were removed from the program standard settings; then, the probes were transformed to their related genomic location. The RMA was employed for normalization of values for the subsequent MA. Then, the difference between each treatment and its control was computed using the LIMMA package. After fitting the data into a linear model, simple empirical Bayes model was used to revise the standard errors. For each contrast in every gene, moderated t-statistic and log-odds of differential expression were calculated. The genes with − 1 ≥ log2 fold change ≥ 1 and *Q-*value cut-off of ≤0.05 were determined as DEGs in each of the four tissues.

#### Integration of significant gene expressions and literature citations for the DEGs

A novel data processing pipeline was proposed in this research integrating different data types to identify promising candidate genes related to salt tolerance in rice (Fig. 4). On one hand, DEGs were integrated in response to salinity stress in rice from both microarray and RNA-Seq technologies. On the other hand, NCBI (NCBI; www.ncbi.nlm.nih.gov) literature was searched to identify the published reports on the salinity tolerance genes in rice. In this research, 111 papers were reviewed and all the reported salinity tolerance-related genes in rice were collected. All the identified genes were classified into four tissues (including shoot, root, seedling, and leaves) (Table S8). Venn diagram (using the R package) was used to compare the overlaps in the detected genes for each tissue using different approaches (including RNA-seq, microarray, and literature review) and the common genes were detected. Finally, salinity tolerance associated meta-QTLs regions were explored to find the DEGs, which are coincided with the meta-QTL positions. For identification of DEGs in meta-QTLs regions, the flanking markers of the identified MQTLs were used to detect the physical intervals for each meta-QTL. Then, the genes located in meta-QTLs regions were found according to the rice genome assembly IRGSP 1.0.

#### Functional annotation and pathway analysis

Enrichment analysis of the DEGs were performed using the AgriGO public web tool [74]. The over-represented GO terms were filtered in the three main categories including the “Biological Process”, “Molecular Function” and “Cellular Component” using the Fisher’s exact test (Q-value < 0.05) and were corrected by the False Discovery Rate (FDR) method at *p* < 0.05.

#### Identification of salinity tolerance-related candidate genes in the meta-QTL regions

The genes observed at least by two approaches (from the three applied methodologies including RNA-seq, microarray and literature review) were called as common genes in this paper.The common genes were sought in the salinity tolerance associated meta-QTLs regions to find the potential candidate genes. The potential candidate genes located on the hotspot-regions overlying original QTLs for both yield components and ion homeostasis traits were assumed as promising candidate genes (Fig. 4).

#### Plant growth and salt stress treatment

Seeds of FL478 as the salt tolerant rice (*Oryza sativa* L.) genotype were provided from International Rice Research Institute (IRRI). Seeds sterilization and germination, as well as plant growth conditions were performed as previously described [31]. Root and shoot samples of 21-days-old treated seedlings with 150 mM NaCl were collected 24 h after inception of the salt stress, instantly put in liquid nitrogen and kept at − 80 °C until RNA extraction.

#### RNA extraction and cDNA library synthesis

Total RNA extraction was performed by the RNeasy Plant kit (Qiagen) from 100 mg of shoot and root tissues. Integrity and quality of RNA samples was inspected using a NanoDrop ND-1000® spectrophotometer and agarose gel electrophoresis. The cDNA library synthesis was done using iScriptTM cDNA synthesis kit (BioBasic) consistent with the manufacturer’s instructions.

#### Validation of salinity tolerance-related candidate genes by qRT-PCR assay

A total of 15 genes from the list of possible candidate genes were randomly nominated in each tissue (Table S6) for validation by quantitative real-time PCR (RT-qPCR). Specific primer pairs for each gene (Table S9 for list primer) were designed by Oligo 7.0 (National Bioscience Inc., Plymouth, USA). The qRT-PCR with three independent biological replicates was done by a LightCycler® 96 Real-Time PCR System (Roche Life Science, Germany) and SYBR Premix without ROX based on manufacturer’s protocol. Actin gene of rice (OS03G0836000) was employed as a suitable inner control gene. Transcript levels of nominated genes from three biological replicates were computed as 2- ΔΔCt [75].

## Supplementary information

**Additional files 1: Table S1.** List of the QTL mapping studies used for meta-QTL analysis for traits associated with the salt tolerance in rice. **Table S2.** The summary of the original QTLs related to salt tolerance traits. **Table S3.** The Summary of the original QTLs related to salinity tolerance included in the meta–analysis. **Table S4.** The consensus QTLs of 32 traits identified by meta–analysis in rice. **Table S5.** The original microarray datasets selected for meta-analysis of rice under salinity stress. **Table S6.** The list of possible candidate genes in the meta-QTL regions (The asterisk on meta position column, represents promising genes located in the hotspot positions). **Table S7.** The list of publicly accessible RNA-seq datasets was used in this study. **Table S8.** The list of reported salinity tolerance related genes in rice based on the literature review, classified into four tissues (including shoot, root, seedling, and leaves) in 4 sheets. **Table S**9. List of primers used for qRT-PCR analysis. **Fig. S1.** Number of the original QTLs that are associated with each salt tolerance related trait (Traits along with their abbreviations are provided in Table S2). **Fig. S2.** Number of the original QTLs related to the salt tolerance in each chromosome of rice. **Fig. S3.** Number of differentially expressed genes (DEGs) identified by RNA-seq meta-analysis in four tissues (including shoot, root, seedling, and leaves). **Fig. S4.** Number of differentially expressed genes (DEGs) identified by microarray meta-analysis in four tissues (including shoot, root, seedling and leaves). **Fig. S5.** GO term assignment of the identified DEGs located in the meta-QTL positions to three main categories of cellular component, molecular function, and biological process. **Fig. S6.** Graph illustrating of the melt curves from qRT-PCR of the selected potential candidate genes in FL478.

## Data Availability

Accession codes: All primary sequence read data has been deposited in NCBI database under BioProject ID: PRJNA493951 and PRJNA493923. All data supporting the conclusions of this article are provided within the article and its supplementary (Additional file 1: Table S1, Table S2, Table S3, Table S4, Table S5, Table S6, Table S7, Table S8, Table S9, Fig. S1, Fig. S2, Fig. S3, Fig. S4, Fig. S5, Fig. S6).

## Abbreviations

DEGs: Differentially expressed genes
QTL: Quantitative trait locus
MA: Meta-analysis
ROS: Reactive oxygen species
KLV: K^+^ in leaf vegetative
NS: Shoot sodium concentration
NKS: Ratio of the shoot sodium and potassium concentration
KS: Shoot potassium concentration
RN: Root Na^+^ concentration
SIS: Salt injury score
QGW: 1000-grain weight (g)
DF: Days to flowering
NFS: Number of fertile spikelets
BP: Biological Processes
GO: Gene Ontology
MF: Molecular Function
CC: Cellular Component
M-QTL: Meta-QTL
CI: Confidence Interval
LOD: Logarithm of the odds ratio
AIC: Akaike Information Criterion
