# Supplementary material for: Salt tolerance involved candidate genes in rice: an integrative meta-analysis approach
Source: BMC Plant Biol. 2020 Oct 1;20:452. doi: 10.1186/s12870-020-02679-8 (PMC7528482; doi:10.1186/s12870-020-02679-8)
Supplement: Supplementary file 1 — Additional files 1: Table S1. List of the QTL mapping studies used for meta-QTL analysis for traits associated with the salt tolerance in rice. Table S2. The summary of the original QTLs related to salt tolerance traits. Table S3. The Summary of the original QTLs related to salinity tolerance included in the meta–analysis. Table S4. The consensus QTLs of 32 traits identified by meta–analysis in rice. Table S5. The original microarray datasets selected for meta-analysis of rice under salinity stress. Table S6. The list of possible candidate genes in the meta-QTL regions (The asterisk on meta position column, represents promising genes located in the hotspot positions). Table S7. The list of publicly accessible RNA-seq datasets was used in this study. Table S8. The list of reported salinity tolerance related genes in rice based on the literature review, classified into four tissues (including shoot, root, seedling, and leaves) in 4 sheets. Table S9. List of primers used for qRT-PCR analysis. Fig. S1. Number of the original QTLs that are associated with each salt tolerance related trait (Traits along with their abbreviations are provided in Table S2). Fig. S2. Number of the original QTLs related to the salt tolerance in each chromosome of rice. Fig. S3. Number of differentially expressed genes (DEGs) identified by RNA-seq meta-analysis in four tissues (including shoot, root, seedling, and leaves). Fig. S4. Number of differentially expressed genes (DEGs) identified by microarray meta-analysis in four tissues (including shoot, root, seedling and leaves). Fig. S5. GO term assignment of the identified DEGs located in the meta-QTL positions to three main categories of cellular component, molecular function, and biological process. Fig. S6. Graph illustrating of the melt curves from qRT-PCR of the selected potential candidate genes in FL478. [file 12870_2020_2679_MOESM1_ESM.zip › Table S5.docx]

| Developmental stages | Tissue | Genotype | Type | Technology | Accession | Seri | No |
| --- | --- | --- | --- | --- | --- | --- | --- |
| Seedling | Leaves, Roots | IR64 | cel | Affymetrix | [GSE58603](http://www.ncbi.nlm.nih.gov/geo/query/acc.cgi?acc=GSE58603) | [GPL2025](http://www.ncbi.nlm.nih.gov/geo/query/acc.cgi?acc=GPL2025) | 1 |
| Seedling | Seedling | Nipponbare and Pokkali | txt | ALLIGNMENT | [GSE48395](http://www.ncbi.nlm.nih.gov/geo/query/acc.cgi?acc=GSE48395) | [GPL17380](http://www.ncbi.nlm.nih.gov/geo/query/acc.cgi?acc=GPL17380) | 2 |
| Seedling | Shoot- Root | Nipponbare | txt | ALLIGNMENT | GSE20746 | [GPL6864](http://www.ncbi.nlm.nih.gov/geo/query/acc.cgi?acc=GPL6864) | 3 |
| Seedling | Whole seedlings | CSR27, MI48, | cel | Affymetrix | GSE16108 | [GPL2025](http://www.ncbi.nlm.nih.gov/geo/query/acc.cgi?acc=GPL2025) | 4 |
| Seedling | Root | FL478, Pokkali and IR63731, IR29 | cel | Affymetrix | GSE14403 | [GPL2025](http://www.ncbi.nlm.nih.gov/geo/query/acc.cgi?acc=GPL2025) | 5 |
| Seedling | Shoot | IR63731 and IR29  Agami and M103 | txt | Affymetrix | GSE4438 | [GPL2025](http://www.ncbi.nlm.nih.gov/geo/query/acc.cgi?acc=GPL2025) | 6 |
| Seedling | Shoot | FL478 and IR29 | txt | Affymetrix | GSE3053 | GPL2025 | 7 |
| Seedlings | Leaf | Oryza sativa | cel | Affymetrix | GSE21651 | GPL2025 | 8 |
| Sseedlings | Seedlings | Oryza sativa | txt | Agilent | GSE79043 | GPL8852 | 9 |
